# Statin drugs to reduce breast cancer recurrence and mortality

**DOI:** 10.1186/s13058-018-1066-z

**Published:** 2018-11-20

**Authors:** Colin H. Beckwitt, Adam Brufsky, Zoltán N. Oltvai, Alan Wells

**Affiliations:** 1 Department of Pathology, University of Pittsburgh, Pittsburgh, 15231 PA USA; 2 Department of Bioengineering, University of Pittsburgh, Pittsburgh, 15231 PA USA; 3 Department of Computational and Systems Biology, University of Pittsburgh, Pittsburgh, 15231 PA USA; 4 University of Pittsburgh Cancer Institute, University of Pittsburgh, Pittsburgh, PA 15231 USA; 5 Pittsburgh VA Health System, Pittsburgh, 15240 PA USA; 6 Magee-Women’s Hospital of Pittsburgh, 300 Halket St., Pittsburgh, 15213 PA USA

**Keywords:** Breast cancer, Statins, Metastasis, Lipophilicity, Prenylation, Secondary prevention

## Abstract

Epidemiologic studies have, variably, shown the concomitant use of statin drugs to be beneficial to cancer outcomes. Statin drugs have been FDA approved for three decades for the treatment of high cholesterol and atherosclerotic coronary artery disease and are widely used. This has engendered studies as to their influence on concomitant diseases, including cancers. In this context, statin use has been correlated, variably, with a decrease in deaths from breast cancer. However, there is no extant model for this effect, and the extent of efficacy is open to question.

The overarching goal of this article is to communicate to the reader of the potential of statins to reduce breast cancer progression and mortality. This is the use as a secondary prevention measure, and not as a therapy to directly counter active cancer. First, salient aspects of statin pharmacology, as relates to cardiovascular disease, will be discussed. Second, the basic and clinical research studies that investigate statin usage in breast cancer will be presented. Additionally, statin effects in other cancer types will be included for context. Finally, proposals for future basic and clinical research studies to determine the role of statins in breast cancer management will be presented.

**Electronic supplementary material:**

The online version of this article (10.1186/s13058-018-1066-z) contains supplementary material, which is available to authorized users.

## Background

Statins reduce blood cholesterol by inhibiting the rate-limiting step in the mevalonate pathway: HMG-CoA reductase (HMGCR). While this decreases the cholesterol carried in the blood, the mechanistic aspects also affect cell signaling, in a manner that could directly impact cell proliferation, and thereby cancer cells. Mevalonate pathway inhibition reduces the abundance of farnesyl pyrophosphate (FPP) and geranylgeranyl pyrophosphate (GGPP), isoprenyl groups that critically modify small signaling G proteins involved in cell proliferation, migration, and survival pathways. The influence of the statin drugs on protein prenylation is not limited to just influencing cardiovascular pathologies but can be applied in the context of other diseases, particularly cancer in which progression depends on increased migration, survival, and ultimately proliferation.

Relevant to this review, epidemiologic studies have, variably, shown the concomitant use of statin drugs to be beneficial to cancer outcomes. The aims of this article, based on literature findings both in vivo and in vitro, are to review recent studies related to statin effects on breast cancer from both a clinical and basic aspect, so as to: 1) form a consensus from the existing literature regarding the mechanisms by which statins influence cancer; and 2) establish a model of how these mechanisms influence cancer incidence, recurrence, and mortality. Existing studies suggest that statins can reduce tumor cell growth and survival, which translates to a clinical benefit of statins on reducing cancer recurrence and mortality, with limited effects on incidence of primary cancer.

### Statin pharmacology and cardiovascular usage

**Fig. 1:**
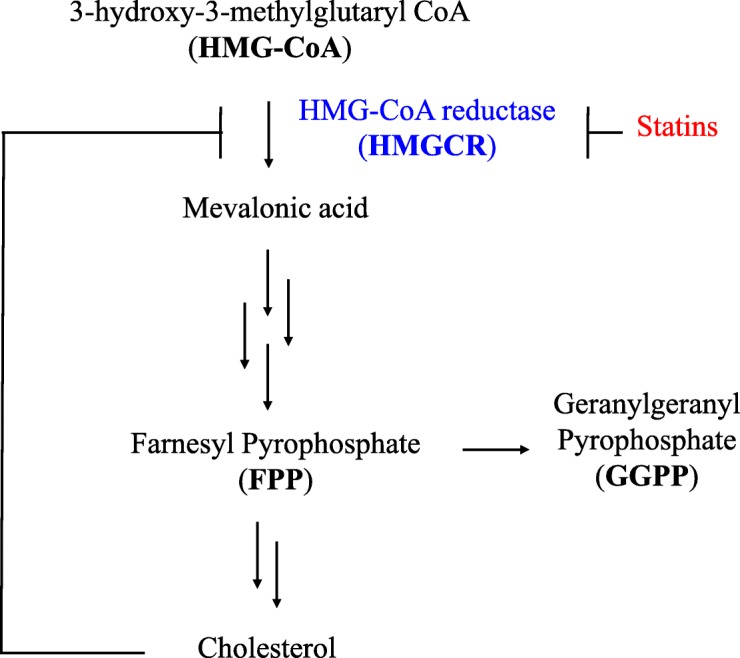
The cholesterol biosynthesis pathway. Statins block HMG-CoA reductase (*HMGCR*, shown in *blue*) to shut down the cholesterol biosynthetic pathway. In addition to cholesterol, other downstream mediators are affected, such as farnesyl pyrophosphate (*FPP*) and geranylgeranyl pyrophosphate (*GGPP*)

The first statins were isolated as potent competitive inhibitors of HMG-CoA reductase (HMGCR) by Akira Endo [1], motivated by previous studies that suggested HMGCR was an important node for the regulation of de novo cholesterol synthesis (Fig. 1) [2]. After success in clinical trials, lovastatin became the first FDA-approved statin in 1987 [1, 3]. Subsequently, six other statins have garnered and maintained FDA approval: atorvastatin, fluvastatin, pitavastatin, pravastatin, rosuvastatin, and simvastatin (Additional file 1) [1]. While all statins share the same pharmacophore, their structural characteristics guide their binding affinity to HMGCR [4] (Additional file 2). The most biologically relevant molecules affected by inhibition of HMGCR (Fig. 1) are downstream of mevalonate—cholesterol, farnesyl pyrophosphate (FPP), and geranylgeranyl pyrophosphate (GGPP).

The seven FDA-approved statins have similar, but not identical, pharmacokinetics (Additional file 3). Statins are classified as lipophilic or hydrophilic depending on their partition coefficients, with hydrophilic statins exhibiting Log D values less than zero and lipophilic statins exhibiting Log D values greater than zero [5]. Statin uptake into cells occurs through carrier-mediated transport, most commonly by OATP1B1 with lesser contributions from OATP1B3, 1A2, and 2B1, or for lipophilic statins through passive transport across the membrane [6, 7]. Importantly, except for hepatocytes, most cell types do not express these transporters at meaningful levels [6]. This means lipophilic statins can distribute into the cells in extrahepatic tissues by diffusion across the cell membrane and will affect the cholesterol synthesis pathway in most cells, whereas hydrophilic statins are basically inert to most cells [8]. The implications for this review is that only the lipophilic statins would be expected to affect breast cancer cells if the effects on the cancer cells are direct, rather than indirect via cholesterol levels.

The primary action of statins is to reduce circulating lipids, most specifically cholesterol packaged in low density lipoprotein (LDL) particles [9]. Statins accomplish this by acting mainly in hepatocytes, the primary site of cholesterol biosynthesis [10]. Statin binding to HMGCR prevents it from converting HMG-CoA into mevalonate, which reduces the intracellular cholesterol concentration [11]. Cholesterol starvation in hepatocytes increases expression of LDLR, in turn facilitating uptake of LDL particles, which reduces blood cholesterol [12]. Thus, statins both reduce new cholesterol synthesis and enhance hepatic uptake of existing, circulating cholesterol. This aspect would represent an indirect effect on cancer cells via slightly decreasing the levels of circulating nutrient.

In addition to modifying lipid homeostasis, statins exert pleotropic effects on other aspects of cardiovascular pathobiology. Cell-mediated effects stem from reductions in cellular signaling from small G proteins through reducing the availability of FPP and GGPP [9]. For example, in endothelial cells, decreased activation of Rho enhances expression of endothelial nitric oxide synthase (eNOS), which protects endothelial function and impedes cardiovascular disease progression [13]. Thus, if the statin could gain entry (as in lipophilic statins) tumor cells would be affected.

Statin usage in the US has increased dramatically over the past several decades. Between 2003 and 2012, the percentage of adults in the US taking statins increased from 18 to 26% [14]. The clinical potency of statins is determined by the measurement of blood LDL cholesterol (LDL-C) in mg/dL [15]. Statin regimens that achieve < 30%, 30–50%, or > 50% reductions in LDL-C are labeled low-, moderate-, and high-intensity statin therapy, respectively [16] (Additional file 4). Statins are the first line of treatment for hyperlipidemia and atherosclerotic cardiovascular disease (ASCVD) and are also used originally as secondary and now as primary preventative therapy for patients at a high risk for developing cardiovascular disease.

## Statin usage in cancer

This background sets the stage for the unexpected effects statins may have on cancer. As their use is widespread, there is a large overlap between people on chronic or long-term statin therapy and those with cancer. Of relevance to the present review are the basic and clinical research studies that have investigated the roles of statin drugs in cancer incidence, progression, and mortality.

### Clinical research studies

While few prospective clinical trials have been conducted to investigate statin therapy in cancer patients, multiple retrospective cohort studies and meta-analyses have been published. Several hypotheses motivate these studies. First, since statins lower LDL-C, this may influence carcinogenesis and tumor progression, as rapidly dividing cells require increased cholesterol for cell membrane synthesis [17]. In addition, the influence of statins on reducing prenylation may reduce signaling through pathways involved in carcinogenesis and cancer progression [18]. Finally, statins reduce the secretion of pro-inflammatory cytokines, which have been shown to drive carcinogenesis and metastasis [19]. Since these three factors influence both early (carcinogenesis) and late (metastasis) stages of cancer progression, clinical studies have queried the influence of statins on cancer incidence, recurrence, and mortality (Table 1). While data on many cancer types will be presented, the primary cancer type discussed will be breast cancer.

**Table 1:** The influence of statin therapy on cancer incidence, recurrence, and mortality

Cancer type	The effect of statin therapy on cancer		
	Incidence	Recurrence	Mortality
Breast	↔	↔ (Hydrophilic statins) ↓ (All or lipophilic statins)	↔ (Hydrophilic statins) ↓ (All or lipophilic statins)
Prostate	↔	↔ (Radical prostatectomy) ↓ (Radiotherapy)	↓ (All, lipophilic, or hydrophilic statins)
Lung	↔	↔ (Pravastatin) ↓ (All statins)	↔ (Pravastatin) ↓ (All statins)
Colorectal	↔	↓ (All statins)	↓ (All statins)
Primary liver	↓ (All Statins)	↓ (All statins or pravastatin)	↓ (All statins or pravastatin)

The influence of statins on cancer incidence, recurrence, and mortality for breast, prostate, lung, colorectal, and primary liver cancer. ↔ = no effect, ↓ = reduction. All statins, hydrophilic statins, and lipophilic statins refer to studies that were conducted examining patients taking any statin, any hydrophilic statin, or any lipophilic statin, respectively

### Statin influence on cancer incidence

Many studies have investigated the influence of statin therapy on the risk for developing cancer, with conflicting results. The CARE trial in 1996 published one of the first associations between statins and cancer incidence, in which pravastatin users demonstrated a statistically significant increase in breast cancer risk [20]. Similarly, the PROSPER trial in 2002 showed a significant increase in new cancer diagnoses in patients receiving pravastatin compared to those on placebo [21]. In contrast, some studies have demonstrated a decreased risk of developing cancer while taking statins. Shi and colleagues demonstrated a reduction in the risk of liver cancer among users of any statin drug, possibly explained by the relatively higher local concentrations of statins in the liver [22]. In addition, as both lipophilic and hydrophilic statins gain access to hepatocellular carcinoma cells, this may represent a special case.

The largest studies have focused on colorectal, lung, prostate, and breast cancer. In colorectal cancer, large retrospective cohort studies demonstrated no influence of statins on disease incidence [23, 24]. In lung cancer, the largest cohort and meta-analysis studies have shown no effect of statins on cancer incidence [25, 26]. In prostate cancer, the majority of studies that have investigated the influence of statins on incidence find no association, especially when correcting for the rate of prostate-specific antigen (PSA) screening [27, 28]. These studies argue for statins not being contributory to carcinogenesis.

In breast cancer, no association between statin usage and increased cancer risk has been reported in the literature [29]. Meta-analyses conducted by Undela et al. [30] and Bonovas et al. [31] show no significantly different incidence of invasive breast cancer in statin users across multiple studies. Other meta-analyses that investigated the incidence of a multitude of cancers among statin users also show no difference in breast cancer incidence with statin therapy [32, 33]. Similarly, in the Nurses’ Health Study, no differences in invasive breast cancer incidence were observed between statin never users, former users, or current users [34]. Finally, the Women’s Health Initiative demonstrated no difference in incidence of breast cancer between statin users and non-users; however, a decrease in the diagnosis of advanced stage breast cancer was seen in users of lipophilic statins [35]. These analyses echo other carcinomas in finding no effect on mammary carcinogenesis, though the decrease in advanced breast cancers may argue for reduction of tumor progression.

### Statin influence on breast cancer recurrence and mortality

Fewer studies have investigated the influence of statins on cancer recurrence. While a consistent decrement in recurrence occurs in liver cancer [36], the findings for most other cancers are equivocal. In breast cancer, however, the majority of clinical evidence supports a protective effect of statins on reducing recurrence [29]. The benefits of statins on reducing breast cancer recurrence appears to be strongest in younger patients, suggesting a longitudinal influence of statin therapy [37]. Manthravadi and colleagues demonstrated a 36% reduction in the risk of breast cancer recurrence in patients taking lipophilic statins [38]. The most convincing evidence in the literature was a population study of all stage I–III breast cancer patients in Denmark conducted by Ahern and colleagues [39]. They found a 10% reduction in breast cancer recurrence among women who were prescribed a lipophilic statin (most commonly simvastatin) but a risk identical to those not on statins among women who were prescribed a hydrophilic statin [39]. These data in aggregate suggest lipophilic statins, and not hydrophilic statins, reduce the risk of breast cancer recurrence, which may indicate a role for statins as agents to impede this mortal stage of tumor progression and secondarily implicate a non-cholesterol effect such as direct tumor cell reduction of prenylation or an indirect effect via reduced inflammatory cytokine release.

Further supporting the contention that statins impact tumor progression, the majority of published data suggest that statins have a mortality benefit in most cancer types. In primary liver cancer, pravastatin and any statin usage reduces cancer-specific mortality [40–42]. Similarly, in colorectal cancer, statin usage reduces cancer-specific mortality, in particular when used either prior to diagnosis or prior to recurrence [43, 44]. In lung cancer, retrospective studies have shown that statins reduce cancer-specific mortality [45, 46]. LUNGSTAR, a phase III randomized clinical trial, found no influence of pravastatin on the survival of patients with small-cell lung carcinoma [47]. This result is partly expected if the mechanism is tumor-cell intrinsic, as pravastatin is hydrophilic, meaning its uptake by tumor cells is limited. In prostate cancer, statin usage significantly reduces cancer-specific and all-cause mortality, particularly when used after diagnosis [48, 49].

In breast cancer, the majority of studies suggest statin usage significantly reduces breast cancer-specific mortality (BCSM). A meta-analysis by Liu et al. demonstrated a 43% reduction in BCSM in women with breast cancer, which was confined to those taking lipophilic statins [50]. A nationwide cohort study in Finland by Murtola and colleagues demonstrated a 46% and 54% reduction in BCSM for pre- and post-diagnosis statin users, respectively [51]. A nationwide cohort study in the UK by Cardwell et al. demonstrated a 16% reduction in BCSM considering all statins, but a greater reduction in patients taking simvastatin, a lipophilic statin [52]. Finally, a nationwide cohort study in Scotland by McMenamin et al. demonstrated a 15% reduction in BCSM in pre-diagnosis statin users [53].

Two additional studies provide convincing data for an overall benefit of statins on all site cancer-specific mortality (ASCSM). First, a meta-analysis by Zhong et al. showed a 31% and 23% reduction in ASCSM in pre- and post-diagnosis statin users, respectively [54]. Second, a nationwide cohort study in Denmark demonstrated a 13–17% reduction in ASCSM in pre-diagnosis statin users compared to never-users [55]. These data suggest statin usage can reduce cancer-specific mortality. Coupling this with the foregoing data suggesting statins can decrease breast cancer recurrence leads to the conclusion that statins can reduce the percentage of patients who experience relapse after therapy and thereby increase breast cancer survival.

### Summary

Many clinical studies have demonstrated that statins can influence carcinogenesis and cancer progression. While statins do not affect the incidence of most cancers, they do exert significant benefits on recurrence and survival in many cancer types, including breast cancer. Importantly, statin recurrence and mortality benefits are most strongly seen with lipophilic statins. These clinical studies motivate further investigation of statins in basic research studies and targeted clinical trials to evaluate potential additive or synergistic effects of statins when used with standard therapies.

## Basic research studies

The clinical studies discussed above have been complemented by basic research studies that focus on the mechanisms by which statins exert anti-tumor effects. Statins may affect tumor cells directly in four main ways: 1) growth suppression, 2) apoptosis induction, 3) anti-invasive and anti-metastatic effects, and 4) anti-angiogenic effects. These would be in addition to the indirect effects of lowering available cholesterol. However, the findings that most beneficial effects of statins accrue to those on lipophilic statins suggest direct effects on the tumor cells. Interestingly, the two exceptions wherein even hydrophilic statins present benefit, liver and prostate cancers, further support the direct effect hypothesis as these cancers possess the transporters that allow cellular entry of the hydrophilic statins.

### Statin suppression of cancer cell growth

Previous in vitro studies have demonstrated that statins can halt cancer cell proliferation by inducing G0/G1 or G2/M arrest. Many pathways have been described that contribute to this anti-proliferative effect. First, simvastatin has been shown to induce G1 cell cycle arrest through reduction of CDK4/6 and Cyclin D1 [56]. Additionally, BRCA1 overexpression in breast cancer cells has been shown to sensitize cells to lovastatin treatment through regulation of CDK4/Cyclin D1 [57]. Second, simvastatin, fluvastatin, and lovastatin have been shown to block the CDK2/Cyclin E-mediated G1/S transition [58]. Third, statins block the DNA-binding activity of NF-ĸB and Activator protein-1 (AP-1), which results in decreased transcription of genes that regulate cell proliferation [4]. Fourth, statins have been shown to inhibit proliferation of breast cancer cells by suppressing FPP and GGPP modification and activation of Ras, Rac, and Rho small GTPases [59]. Rho GTPases normally play an important role in p27 degradation, meaning inactivity results in the accumulation of p27 and cell cycle arrest [60]. Similarly, statin-mediated decreases in Ras activity result in decreased downstream signaling through Akt and diminished cell proliferation [61]. Finally, statins have been shown to inhibit DNA methyltransferases, altering gene transcription and reducing cancer cell proliferation [62]. In summary, statins act to suppress cancer proliferation by inducing cell cycle arrest, blocking proliferation signals, and influencing gene transcription.

### Statin induction of cancer cell apoptosis

In addition to suppressing growth of tumor cells, some literature reports that statins can induce apoptosis. Several pathways have been described that contribute to induction of apoptosis. First, statins have been shown to decrease the protein levels of anti-apoptotic proteins such as Bcl-2 and Bcl-xL [63, 64]. Second, statins have also been shown to upregulate the activation of pro-apoptotic molecules such as Bax, Bad, and Caspases 3, 8, and 9 [63–66]. Additionally, statins have been shown to decrease phosphorylation and degradation of Bim in breast cancer cells [67], promoting apoptosis. Third, statins can induce reactive oxygen species (ROS) generation, resulting in an increase in p38 MAPK and activation of apoptotic pathways [68]. Similarly, iNOS-mediated generation of nitric oxide was shown to contribute to simvastatin- and fluvastatin-induced apoptosis in breast cancer cells [66]. Fourth, statins can activate JNK-mediated apoptotic pathways that are independent of p53 activation [69]. Finally, statins can induce calcium-dependent apoptosis through increased mitochondrial uptake of calcium through L-type calcium channels, resulting in the release of cytochrome C [70]. It is suggested that these pro-apoptotic effects are mainly mediated by a reduction in GGPP- rather than FPP-modified proteins [4].

However, other studies demonstrate no influence of statins on apoptosis. For example, simvastatin was shown to decrease proliferation of bladder cancer cells without inducing apoptosis [56]. Moreover, simvastatin at physiologically relevant concentrations was shown to protect osteosarcoma cells from oxidative stress-induced apoptosis by upregulation of Bcl-2 [71]. While atorvastatin treatment could reduce Bcl-2 expression in MDA-MB-231 breast cancer cells, two weeks of neoadjuvant atorvastatin at 80 mg/kg in human breast cancer patients was not found to alter Bax or Bcl-2 expression [69]. Moreover, other studies report minimal effects of statins on inducing cancer apoptosis [72] or a significant influence on apoptosis at concentrations much higher than currently used clinically [4]. Thus, the precise mechanism and magnitude of statin-induced apoptosis of cancer cells remains unclear.

### Statin suppression of angiogenesis

Statins exert biphasic effects on angiogenesis—pro-angiogenic effects at low (nanomolar) concentrations, anti-angiogenic effects at higher (micromolar) concentrations [73]. At low concentrations, statins can induce mobilization and differentiation of endothelial progenitor cells (EPCs) through stimulation of the PI3K-Akt pathway [74]. Similarly, statin treatment can induce proliferation in EPCs through upregulation of cyclin genes and downregulation of the CDK2 inhibitor p27 [75]. Moreover, low statin concentrations can enhance endothelial cell migration and tube formation in a PI3K-Akt- and endothelial nitric oxide synthase (eNOS)-dependent manner [76]. The PI3K-Akt pathways seem to be required for statin-mediated angiogenesis, as blocking PI3K eliminates pro-angiogenic effects of statins [77].

In contrast, at higher concentrations, statins negatively impact angiogenesis through multiple mechanisms. First, statins have been shown to reduce membrane localization of RhoA by blocking its geranylgeranylation, which reduced the tube-forming ability of human endothelial cells in vitro and was reversed with GGPP supplementation [78]. This inhibition of RhoA subsequently suppresses signaling through pathways that require RhoA activity, such as VEGFR, FAK, and Akt [78]. Second, statins decrease the abundance of caveolin-1 in endothelial cells, which reduces VEGFR2-mediated angiogenic signaling in a cholesterol-independent manner [79]. Third, endothelial cells treated with relatively higher doses of statins show a decrease in hypoxia-stimulated secretion of VEGF and expression of VEGFR2 [73]. Finally, simvastatin has been shown to upregulate vascular epithelial cadherin (VE-cadherin), which limits endothelial cell proliferation, migration, and tube forming ability.

In the context of cancer, statins can reduce tumor angiogenesis through several different mechanisms. First, statins can directly affect the endothelial cells to reduce tumor angiogenesis. For example, simvastatin treatment was shown to decrease tumor vascularization in mice at high statin doses [73]. Second, statins can act indirectly on the endothelium by reducing pro-angiogenic or increasing anti-angiogenic protein secretion by tumor cells [80]. Atorvastatin has been shown to reduce circulating VEGF concentrations in human patients with coronary artery disease [81]. Finally, simvastatin and atorvastatin can reduce matrix metalloproteinase (MMP)-9 expression in endothelial cells, which reduces their invasive capability [82]. Moreover, MMP-9 has been shown to be important for releasing the VEGF that is sequestered in breast cancer extracellular matrix (ECM) [83]. Thus, a reduction in MMP-9 impacts both the stimulation for and competency of endothelial cells to initiate angiogenesis.

### Statin suppression of cancer cell invasion and metastasis

Many in vitro studies have suggested statins reduce the invasiveness and metastatic potential of cancer cells through multiple different mechanisms. First, statin treatment destabilizes the cytoskeletal structure of tumor cells in a RhoA/RhoC-dependent manner. Upon statin treatment, Rho delocalizes from the membrane, which causes breakdown of the actin cytoskeleton, loss of actin stress fibers and focal adhesion sites, and cell rounding [84, 85]. Moreover, statin treatment is sufficient to block EGF-mediated RhoA membrane localization and formation of actin stress fibers [86]. Second, statins block adhesion of cancer cells to both ECM proteins by reducing integrin binding activity [87]. Similarly, statins downregulate E-selectin on tumor endothelial cells, which reduces tumor cell adhesion and invasion through an endothelial barrier [88]. Third, statins reduce expression and activity of the pro-migratory proteases MMP-2, MMP-9, and urokinase by inhibiting Ras and Rho activity [84]. Fourth, statin treatment can downregulate the cancer stem cell marker CD44 in breast cancer cells, which reduces cell migration and invasion [89]. Fifth, statins can reduce expression of the transferrin receptor in breast cancer cells, which causes iron starvation and a reduction in tumor invasiveness [90]. Finally, we have shown that E-cadherin can induce tumor cell resistance to statins [91]. This suggests that statins specifically target the cells primed to undergo the epithelial–mesenchymal transition (EMT), invade, and metastasize.

Statins also impact tumor invasion and metastasis in ex vivo and in vivo models. Statins were shown to reduce prostate cancer migration towards human bone marrow stroma without impacting ECM adhesion [92]. Also, lovastatin, atorvastatin, and simvastatin have been shown to reduce metastatic seeding of melanoma and breast cancer cells in the lung and bone [85, 89, 93]. In breast cancer patients receiving 40 mg simvastatin per day for 4–6 weeks before mastectomy, statin treatment was able to decrease post-explant tumor migration [94]. Moreover, simvastatin treatment reduced the activity of Rho associated protein kinase (ROCK) and expression of RhoC, chemokine receptor type 4 (CXCR4), and CD44 [94]. In a sophisticated ex vivo microphysiological model of breast cancer metastasis to the liver, breast cancer outgrowth in response to an inflammatory lipopolysaccharide/EGF stimulus could be suppressed with atorvastatin treatment [95]. Finally, we have shown in mouse models of spontaneous breast cancer metastasis to liver and lung that atorvastatin is able to suppress metastatic proliferation but not the proliferation of the primary tumor [95]. These data suggest that statins preferentially target mesenchymal, metastatic cells.

### Summary

**Fig. 2:**
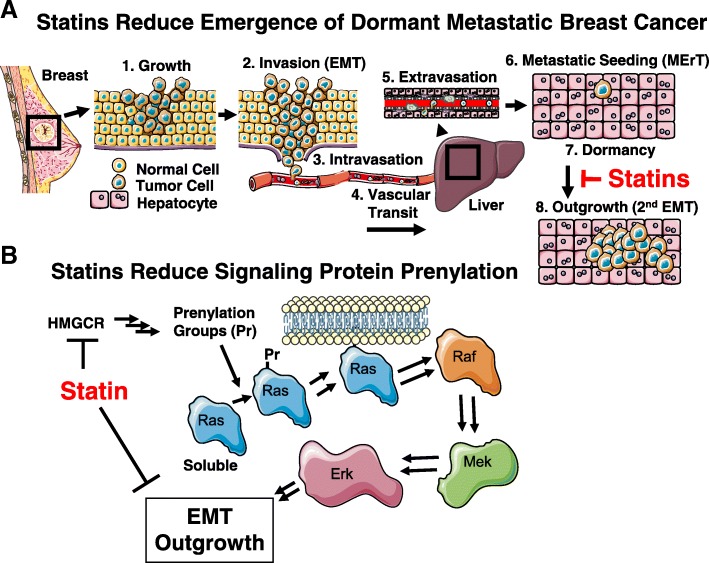
Proposed model for statin action in breast cancer. **a** The breast cancer metastatic cascade. Statins (*red*) block emergence of dormant breast cancer cells at the site of micrometastasis to prevent their emergence to form clinically evident metastases. **b** Statins (*red*) block HMG-CoA reductase (*HMGCR*) to decrease the number of prenylation groups (*Pr*), such as farnesyl pyrophosphate and geranylgeranyl pyrophosphate, available for prenylating small G proteins, such as Ras (shown), Rac, and RhoA. Decreased prenylation reduces membrane tethering of these G proteins, which reduces downstream proliferative and pro-EMT signaling. Drawing made using images from Servier Medical Art [101]

Many basic research studies have shown that statins can reduce tumor cell growth and proliferation by inducing cell cycle arrest. Additionally, some, but not all, studies suggest that statins can induce apoptosis in cancer cells. In addition to affecting tumor cell growth and apoptosis, statins can negatively impact the tumor vasculature through suppression of angiogenesis. While statins exhibit a biphasic effect on angiogenesis, their anti-angiogenic effects are observed at similar doses to those used in studies that demonstrate anti-tumor effects. While statins have been shown to reduce cancer cell migration and invasion in vitro, few studies have demonstrated a reduction in metastasis with statin treatment in vivo. These previous studies motivate the investigation of statins in the context of breast cancer metastasis, in particular dormancy and emergence, which is the main cause of morbidity and mortality in this patient population. We propose a model in which statins block the secondary EMT and outgrowth of tumor cells by preventing Ras, Rac, and RhoA prenylation (Fig. 2).

## Toxicity

An important consideration for repurposing statin drugs for breast cancer treatment is toxicity. Breast cancer patients already receive chemotherapeutic compounds that exhibit varying degrees of toxicities. Fortunately, statins are typically well-tolerated drugs. The most common adverse events associated with statin usage involve muscle tissues, which range in severity from myalgia to fatal rhabdomyolysis [8]. Muscle pain (myalgia) is relatively more common among statin users, with an incidence of approximately 5% [96]. Myopathy, or myositis, only occurs in 0.1% of patients taking statins and involves pain, weakness, and mobility restrictions that often are accompanied by an elevation in serum creatine kinase (CK) to ten times the upper limit of normal [96]. Rarely, myopathy can progress to rhabdomyolysis, which involves CK elevations above 50 times the upper limit of normal and myoglobin accumulation in the blood (myoglobinemia) and urine (myoglobinuria), which can induce renal failure [96]. The incidence of rhabdomyolysis is extremely rare, with an incidence of 3.4 per 100,000 person years and mortality rate of approximately 10% [97]. Rhabdomyolysis incidence is higher in patients taking atorvastatin, lovastatin, and simvastatin, which is due to their extensive metabolism by CYP3A4 and potential for drug–drug interactions [98]. The less common adverse events observed with statin usage involve the liver, pancreas, kidney, and peripheral nerves.

The key concern for breast cancer patients is that the toxicity of statins leads to generalized inflammation. The initiation of inflammatory cascades can awaken dormant micrometastases to outgrow [99, 100]. Thus, higher dose statins may be perversely contrary and promote cancer progression if they cause a cytokine storm or lead to fibrosis of chronic inflammation. As the clinical correlation studies did not account for toxicity of statins, the benefits may be understated.

## Conclusions and future directions

Previous studies have reported mechanistic and pharmacologic insight into the use of statins as anti-tumor agents, but additional questions remain regarding their use in breast cancer. First, the efficacy of statins against breast cancer cells with different molecular subtypes is not yet well understood. The susceptibility of many breast cancer cell lines—hormone receptor-positive, HER2-positive, and triple-negative—should be investigated in order to determine whether molecular subtype plays a role in statin sensitivity. Moreover, patient-derived cells should be tested to validate the cell line data.

Second, the difference in efficacy of statins against primary and metastatic tumor cells should be further investigated. Since the clinical data demonstrate statins selectively reduce breast cancer mortality and recurrence without affecting cancer incidence, this would suggest statins more selectively target metastatic cells. This may result from the evolution of tumor cells between the primary and metastatic tumor or be related to primary tumor drug penetrance. In order to better investigate the differential potency of statins against primary and metastatic tumor cells, paired biopsy samples from clinical patients should be used in in vitro, ex vivo, and in vivo models of breast cancer metastasis. This may reveal the relative importance of intrinsic differences in statin susceptibility of the primary and metastatic cells and the differences in the primary and metastatic microenvironments. Moreover, there may be a difference in statin susceptibility with the site of metastasis.

Finally, it is critical to understand how statins interact with currently used breast cancer therapies, as statin use may either enhance or diminish their efficacy. For example, since statins work by inducing growth arrest and decreasing proliferation, the efficacy of chemotherapeutic agents may be compromised since they are only effective against cycling cells. If chemotherapeutic efficacy is compromised by statins, patients should stop taking their statin during chemotherapy cycles. In contrast, statins have been shown to induce oxidative stress in cancer cells, which may compromise the health of tumor cells and make them more susceptible to cytotoxic drugs. If these findings hold true, patients who don’t normally take statins should supplement their chemotherapy regimen with a lipophilic statin. Finally, since most statins are metabolized by CYP450 isozymes, they may interfere with the metabolism of chemotherapeutic drugs and possibly induce additional adverse events. Examining statins in combination with other chemotherapeutic drugs in ex vivo liver culture systems and in vivo will suggest which drug combinations may result in higher clinical efficacy or toxicity.

Prospective clinical trials should investigate statin use as adjuvant therapy in breast cancer. Since the studies that demonstrate a cancer mortality benefit with statin use are quite large, prospective clinical trials would need to be conducted in a targeted patient population. For example, investigating adjuvant statin therapy in triple-negative or HER2-positive patients would be more feasible than a trial of hormone receptor-positive patients since the former two groups typically relapse earlier. Moreover, patients with nodal metastases or locally invasive primary tumors would be prime candidates for these trials, as they are at the highest risk for metastatic relapse. Patients would be randomized to a lipophilic statin (likely atorvastatin) or placebo and followed longitudinally. These trials would contain defined interim analysis periods to allow patients to crossover from placebo to statin should the statin recurrence or mortality benefits prove significant.

Future basic research studies and clinical trials that build upon the results of past studies have the potential to establish statins as effective and safe agents to delay breast cancer recurrence and mortality. In the short term, studies should address whether current statin users for cardiovascular indications with a history of breast cancer should be uniformly prescribed lipophilic statins. In the long term, clinical trials should address whether statins play a role in adjuvant management of breast cancer in women who do not otherwise require a statin. It is hoped that accomplishing both of these short- and long-term goals will improve the quality of life and survival rates of women with breast cancer.

## Additional files

Additional file 1: Chemical structures of HMG-CoA, compactin, and FDA-approved statins. The chemical structures of HMG-CoA, compactin, the first derived statin, and the seven FDA-approved statin drugs. Dates in parentheses indicate the initial approval date by the FDA, according to their website [102]. Drug structures were drawn using ChemDoodle Sketcher [103]. (PPTX 6215 kb)

Additional file 2: Affinity of HMG-CoA and statins for HMGCR. The affinity (K_d_) or mean inhibitory concentration (K_i_) of HMG-CoA and statins, respectively, for HMGCR. The statin prefixes are used instead of the whole name (e.g., Atorva = Atorvastatin). Affinity and mean inhibitory concentration values are reported in nanomoles (nM) [104–106]. (PPTX 45 kb)

Additional file 3: Pharmacokinetic properties of the seven FDA-approved statins. Absorption, distribution, metabolism, and excretion parameters for each statin are listed. The statin prefixes are used instead of the whole name (e.g., Atorva = Atorvastatin). Times are represented in hours (h). Log D = distribution coefficient at pH 7.4 (higher value means more lipophilic); *T*_*max*_ time after oral dose to maximum serum concentration; *CYP450* cytochrome P450; *T*_*1/2*_ half-life. Data were acquired from Schachter [8], McKenney et al. [5], and Davidson et al. [107]. (PPTX 57 kb)

Additional file 4: High-, moderate-, and low-intensity statin therapy. Statin dosages that achieve plasma reductions in LDL-C of < 30% (low-intensity), between 30 and 50% (moderate-intensity), and > 50% (high-intensity), as tested in randomized control trials (RCTs) and reported in the 2013 American College of Cardiology (ACC) and American Heart Association (AHA) 2013 guidelines [16]. Statin dosages reported in italics are FDA-approved but have not been tested in RCTs. *BID* twice daily, *XL* extended release. (PPTX 51 kb)

## Abbreviations

ASCVD: Atherosclerotic cardiovascular disease
BCSM: Breast cancer-specific mortality
ECM: Extracellular matrix
EMP: Epithelial-Mesenchymal Plasticity
FPP: Farnesyl pyrophosphate
GGPP: Geranylgeranyl pyrophosphate
HMGCR: HMG-CoA reductase
LDL: Low density lipoprotein
LDL-C: LDL cholesterol
LDL-R: Low density lipoprotein receptor
MMP: Matrix metalloproteinase
PCSK9: Proprotein convertase subtiliskin/Kexin type 9
