# Supplementary material for: Statin drugs to reduce breast cancer recurrence and mortality
Source: Breast Cancer Res. 2018 Nov 20;20:144. doi: 10.1186/s13058-018-1066-z (PMC6247616; doi:10.1186/s13058-018-1066-z)
Supplement: Supplementary file 1 — Chemical structures of HMG-CoA, compactin, and FDA-approved statins. The chemical structures of HMG-CoA, compactin, the first derived statin, and the seven FDA-approved statin drugs. Dates in parentheses indicate the initial approval date by the FDA, according to their website [102]. Drug structures were drawn using ChemDoodle Sketcher [103]. (PPTX 6215 kb) [file 13058_2018_1066_MOESM1_ESM.pptx]

## Slide 1
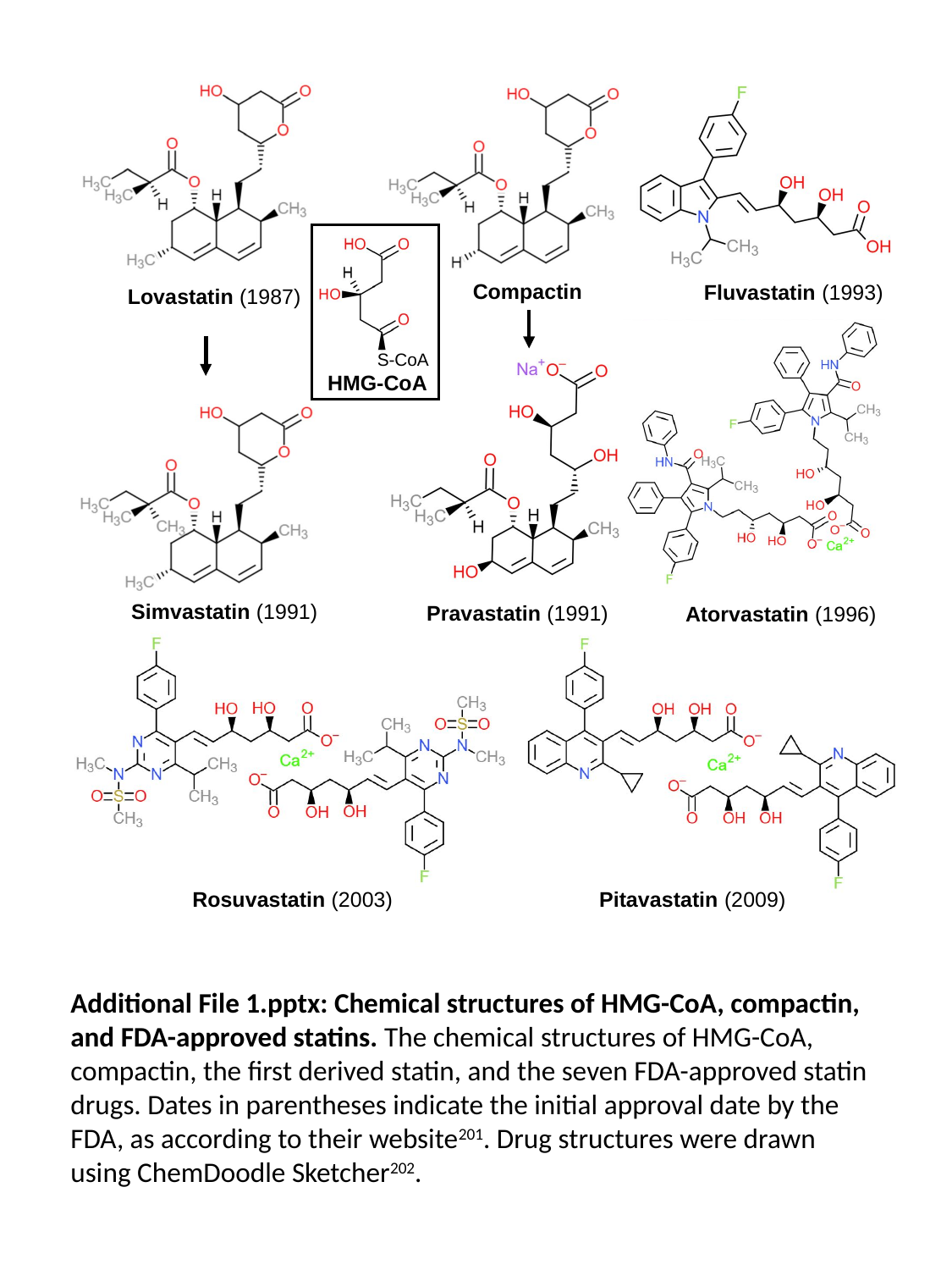

Lovastatin (1987)
Simvastatin (1991)
Fluvastatin (1993)
Atorvastatin (1996)
Compactin
Pravastatin (1991)
Rosuvastatin (2003)
Pitavastatin (2009)
S-CoA
HMG-CoA
Additional File 1.pptx: Chemical structures of HMG-CoA, compactin, and FDA-approved statins. The chemical structures of HMG-CoA, compactin, the first derived statin, and the seven FDA-approved statin drugs. Dates in parentheses indicate the initial approval date by the FDA, as according to their website201. Drug structures were drawn using ChemDoodle Sketcher202.
