# Supplementary material for: Statin drugs to reduce breast cancer recurrence and mortality
Source: Breast Cancer Res. 2018 Nov 20;20:144. doi: 10.1186/s13058-018-1066-z (PMC6247616; doi:10.1186/s13058-018-1066-z)
Supplement: Supplementary file 2 — Affinity of HMG-CoA and statins for HMGCR. The affinity (Kd) or mean inhibitory concentration (Ki) of HMG-CoA and statins, respectively, for HMGCR. The statin prefixes are used instead of the whole name (e.g., Atorva = Atorvastatin). Affinity and mean inhibitory concentration values are reported in nanomoles (nM) [104–106]. (PPTX 45 kb) [file 13058_2018_1066_MOESM2_ESM.pptx]

## Slide 1
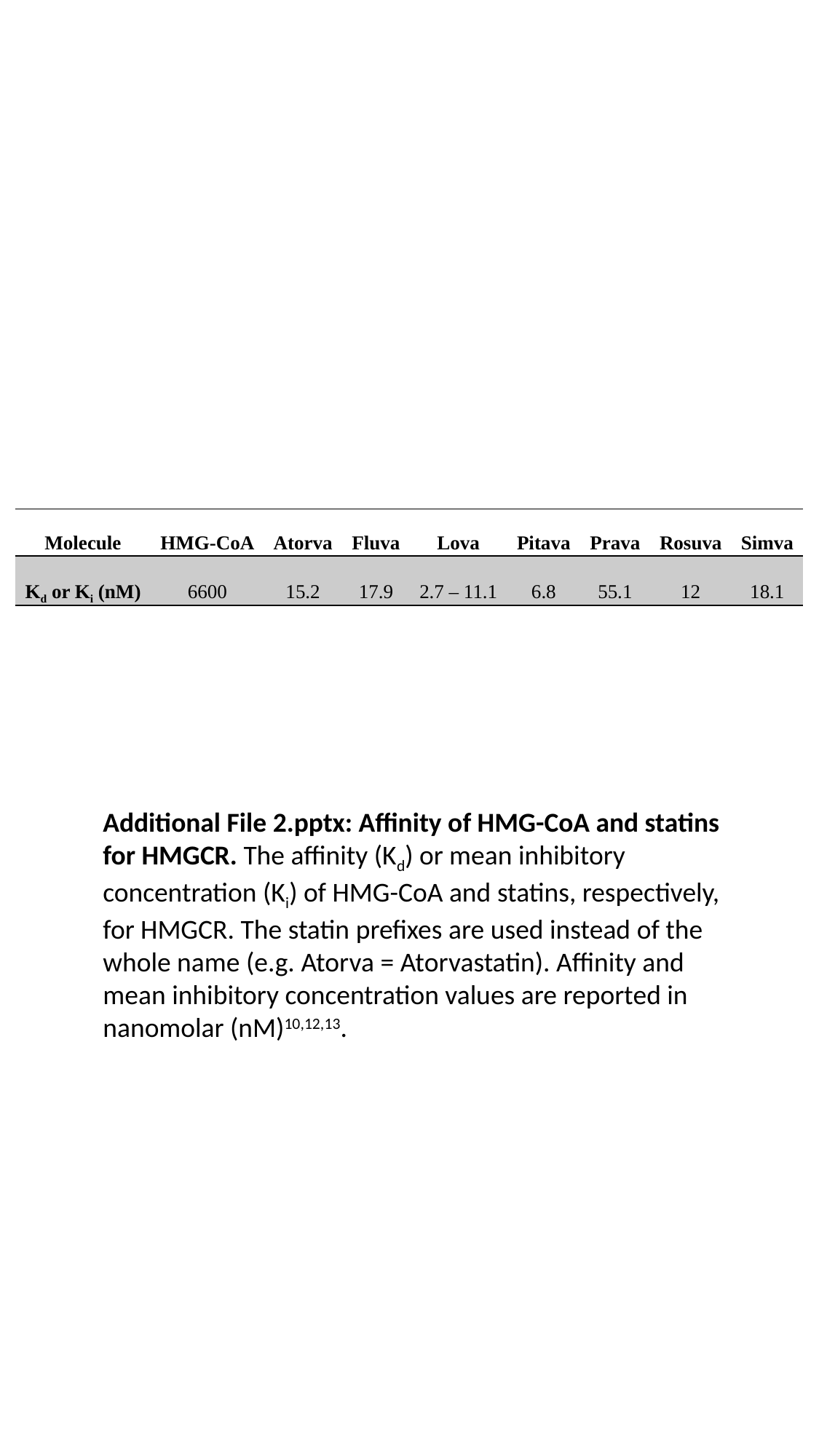

| Molecule | HMG-CoA | Atorva | Fluva | Lova | Pitava | Prava | Rosuva | Simva |
| --- | --- | --- | --- | --- | --- | --- | --- | --- |
| Kd or Ki (nM) | 6600 | 15.2 | 17.9 | 2.7 – 11.1 | 6.8 | 55.1 | 12 | 18.1 |
Additional File 2.pptx: Affinity of HMG-CoA and statins for HMGCR. The affinity (Kd) or mean inhibitory concentration (Ki) of HMG-CoA and statins, respectively, for HMGCR. The statin prefixes are used instead of the whole name (e.g. Atorva = Atorvastatin). Affinity and mean inhibitory concentration values are reported in nanomolar (nM)10,12,13.
