# Supplementary material for: Statin drugs to reduce breast cancer recurrence and mortality
Source: Breast Cancer Res. 2018 Nov 20;20:144. doi: 10.1186/s13058-018-1066-z (PMC6247616; doi:10.1186/s13058-018-1066-z)
Supplement: Supplementary file 3 — Pharmacokinetic properties of the seven FDA-approved statins. Absorption, distribution, metabolism, and excretion parameters for each statin are listed. The statin prefixes are used instead of the whole name (e.g., Atorva = Atorvastatin). Times are represented in hours (h). Log D = distribution coefficient at pH 7.4 (higher value means more lipophilic); Tmax time after oral dose to maximum serum concentration; CYP450 cytochrome P450; T1/2 half-life. Data were acquired from Schachter [8], McKenney et al. [5], and Davidson et al. [107]. (PPTX 57 kb) [file 13058_2018_1066_MOESM3_ESM.pptx]

## Slide 1
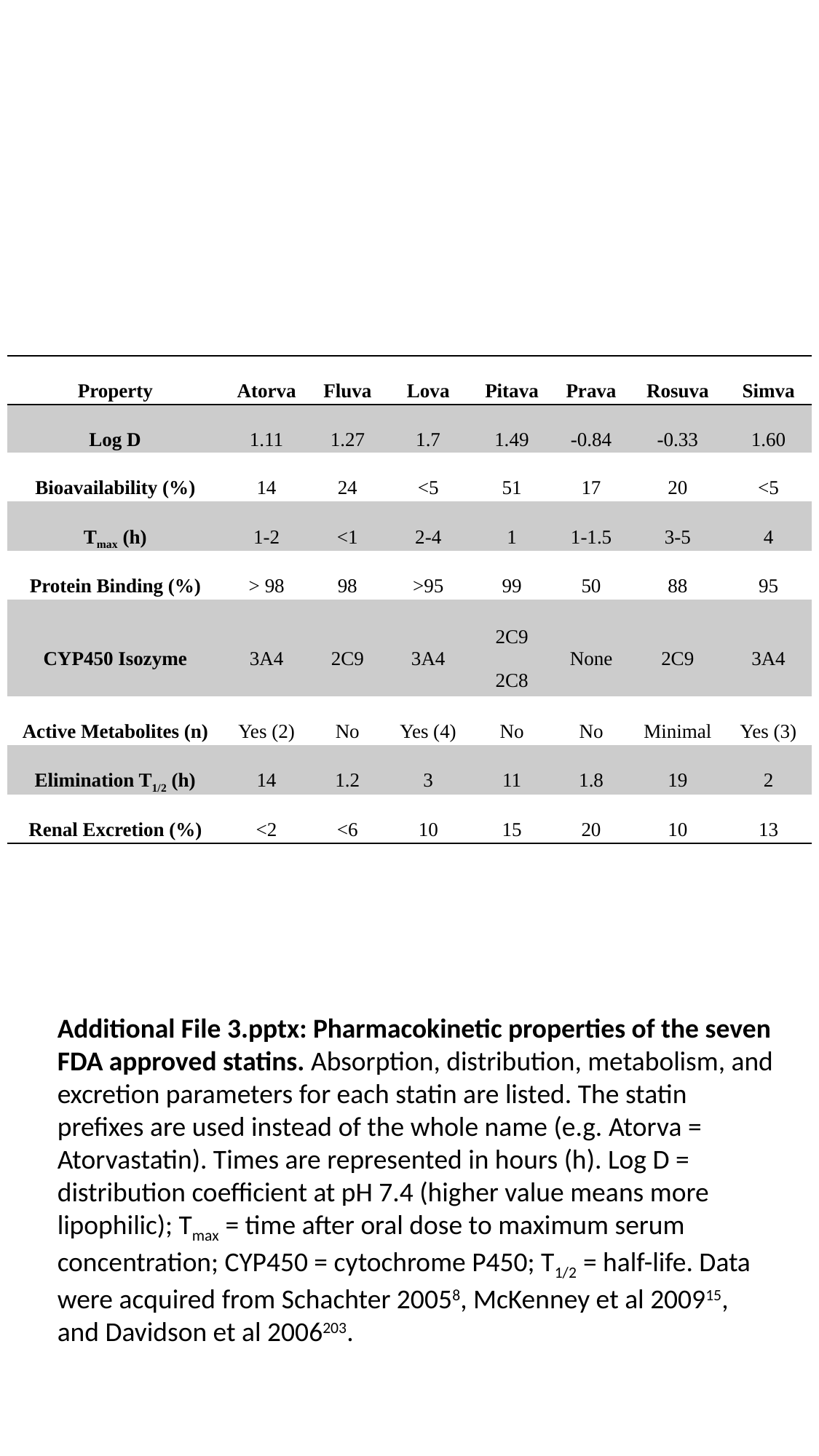

| Property | Atorva | Fluva | Lova | Pitava | Prava | Rosuva | Simva |
| --- | --- | --- | --- | --- | --- | --- | --- |
| Log D | 1.11 | 1.27 | 1.7 | 1.49 | -0.84 | -0.33 | 1.60 |
| Bioavailability (%) | 14 | 24 | <5 | 51 | 17 | 20 | <5 |
| Tmax (h) | 1-2 | <1 | 2-4 | 1 | 1-1.5 | 3-5 | 4 |
| Protein Binding (%) | > 98 | 98 | >95 | 99 | 50 | 88 | 95 |
| CYP450 Isozyme | 3A4 | 2C9 | 3A4 | 2C9 2C8 | None | 2C9 | 3A4 |
| Active Metabolites (n) | Yes (2) | No | Yes (4) | No | No | Minimal | Yes (3) |
| Elimination T1/2 (h) | 14 | 1.2 | 3 | 11 | 1.8 | 19 | 2 |
| Renal Excretion (%) | <2 | <6 | 10 | 15 | 20 | 10 | 13 |
Additional File 3.pptx: Pharmacokinetic properties of the seven FDA approved statins. Absorption, distribution, metabolism, and excretion parameters for each statin are listed. The statin prefixes are used instead of the whole name (e.g. Atorva = Atorvastatin). Times are represented in hours (h). Log D = distribution coefficient at pH 7.4 (higher value means more lipophilic); Tmax = time after oral dose to maximum serum concentration; CYP450 = cytochrome P450; T1/2 = half-life. Data were acquired from Schachter 20058, McKenney et al 200915, and Davidson et al 2006203.
