# Supplementary material for: Statin drugs to reduce breast cancer recurrence and mortality
Source: Breast Cancer Res. 2018 Nov 20;20:144. doi: 10.1186/s13058-018-1066-z (PMC6247616; doi:10.1186/s13058-018-1066-z)
Supplement: Supplementary file 4 — High-, moderate-, and low-intensity statin therapy. Statin dosages that achieve plasma reductions in LDL-C of < 30% (low-intensity), between 30 and 50% (moderate-intensity), and > 50% (high-intensity), as tested in randomized control trials (RCTs) and reported in the 2013 American College of Cardiology (ACC) and American Heart Association (AHA) 2013 guidelines [16]. Statin dosages reported in italics are FDA-approved but have not been tested in RCTs. BID twice daily, XL extended release. (PPTX 51 kb) [file 13058_2018_1066_MOESM4_ESM.pptx]

## Slide 1
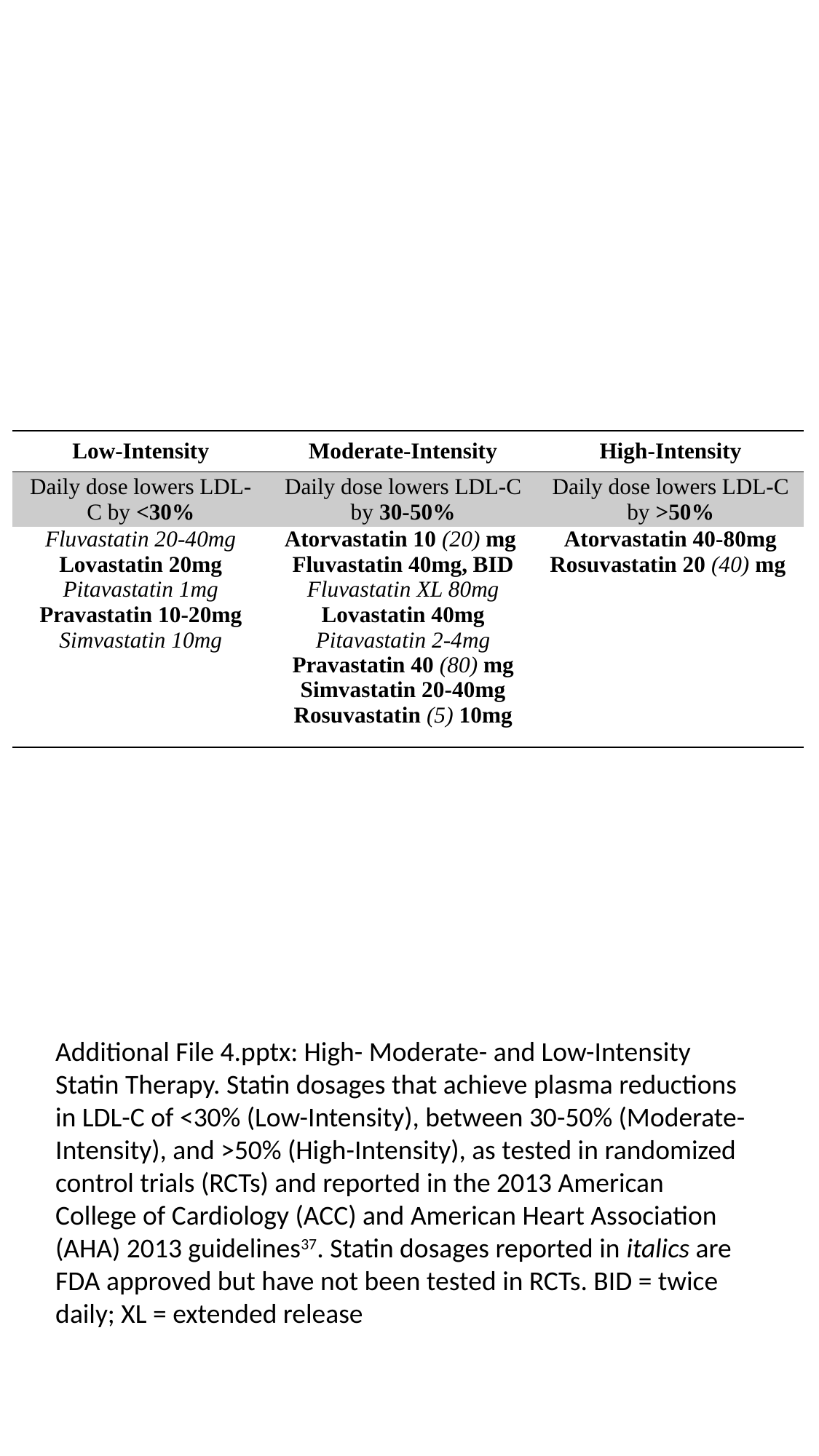

| Low-Intensity | Moderate-Intensity | High-Intensity |
| --- | --- | --- |
| Daily dose lowers LDL-C by <30% | Daily dose lowers LDL-C by 30-50% | Daily dose lowers LDL-C by >50% |
| Fluvastatin 20-40mg Lovastatin 20mg Pitavastatin 1mg Pravastatin 10-20mg Simvastatin 10mg | Atorvastatin 10 (20) mg Fluvastatin 40mg, BID Fluvastatin XL 80mg Lovastatin 40mg Pitavastatin 2-4mg Pravastatin 40 (80) mg Simvastatin 20-40mg Rosuvastatin (5) 10mg | Atorvastatin 40-80mg Rosuvastatin 20 (40) mg |
Additional File 4.pptx: High- Moderate- and Low-Intensity Statin Therapy. Statin dosages that achieve plasma reductions in LDL-C of <30% (Low-Intensity), between 30-50% (Moderate-Intensity), and >50% (High-Intensity), as tested in randomized control trials (RCTs) and reported in the 2013 American College of Cardiology (ACC) and American Heart Association (AHA) 2013 guidelines37. Statin dosages reported in italics are FDA approved but have not been tested in RCTs. BID = twice daily; XL = extended release
